# Immune and oxidative stress disorder in ovulation-dysfunction women revealed by single-cell transcriptome

**DOI:** 10.3389/fimmu.2023.1297484

**Published:** 2023-12-04

**Authors:** Lingbin Qi, Yumei Li, Lina Zhang, Shuyue Li, Xunyi Zhang, Wanqiong Li, Jiaying Qin, Xian Chen, Yazhong Ji, Zhigang Xue, Bo Lv

**Affiliations:** ^1^ Reproductive Medical Center, Department of Gynecology and Obstetrics, Tongji Hospital, Tongji University School of Medicine, Shanghai, China; ^2^ Department of Assisted Reproduction, Xiangya Hospital, Central South University, Changsha, China; ^3^ Shenzhen Key Laboratory of Reproductive Immunology for Peri-implantation, Shenzhen Zhongshan Institute for Reproductive Medicine and Genetics, Shenzhen Zhongshan Obstetrics and Gynecology Hospital (formerly Shenzhen Zhongshan Urology Hospital), Shenzhen, China

**Keywords:** single-cell RNA sequencing, ovulation dysfunction, immune cell disorder, conventional dendritic cell, oxidative stress

## Abstract

**Introduction:**

Ovulation dysfunction is now a widespread cause of infertility around the world. Although the impact of immune cells in human reproduction has been widely investigated, systematic understanding of the changes of the immune atlas under female ovulation remain less understood.

**Methods:**

Here, we generated single cell transcriptomic profiles of 80,689 PBMCs in three representative statuses of ovulation dysfunction, i.e., polycystic ovary syndrome (PCOS), primary ovarian insufficiency (POI) and menopause (MENO), and identified totally 7 major cell types and 25 subsets of cells.

**Results:**

Our study revealed distinct cluster distributions of immune cells among individuals of ovulation disorders and health. In patients with ovulation dysfunction, we observed a significant reduction in populations of naïve CD8 T cells and effector memory CD4 T cells, whereas circulating NK cells and regulatory NK cells increased.

**Discussion:**

Our results highlight the significant contribution of cDC-mediated signaling pathways to the overall inflammatory response within ovulation disorders. Furthermore, our data demonstrated a significant upregulation of oxidative stress in patients with ovulation disorder. Overall, our study gave a deeper insight into the mechanism of PCOS, POI, and menopause, which may contribute to the better diagnosis and treatments of these ovulatory disorder.

## Introduction

In recent decades, female infertility has become an increasing global concern. Knowledge of human reproduction has revealed that immune disorders can affect female fertility at multiple levels (1). Immune disorders affect hormonal glandular functions, such as thyroiditis, resulting in hyperprolactinemia that promotes ovulation dysfunction (2). Meanwhile, it has been reported that antibodies produced by immune cells are associated with high risk of infertility (3). Clinical evidence has confirmed that autoimmune diseases play a critical role in fertility decreasing (4). Ovulation disorder is the most frequent cause of female infertility and is present in approximately 40% of infertile women (5). The most common reasons for the decrease in ovulation function are polycystic ovary syndrome (PCOS), primary ovarian insufficiency (POI), and aging-induced ovarian reserve loss such as menopause (MENO). Although the immune cell types involved in these ovarian disorders are partially known, the essential immune subclusters, their transcriptomic characteristics, and changes in signaling pathways remain unclear (6–8). Sorting immune cells from peripheral blood using flow cytometry in bulk is unable to capture natural transcriptome characteristics. In addition, there still has no specific drugs for the treatment of either PCOS or POI (9, 10). Menopause, as a result of women losing their reproductive capacity and suffering from endocrine disorders with aging, also lacks immune cell data. Therefore, further studies are needed to understand specific anovulation-associated pathogenic factors.

Understanding immune alterations in peripheral blood is of paramount importance for unraveling the pathogenesis of ovulatory disorders. Comparative analysis of scRNA-seq datasets among individuals with varying health conditions, including PCOS, POI, and menopause, revealed notable changes in subsets of well-known cell types. It is widely acknowledged that naive T cells serve as precursors for effector and memory T cell subsets. Previous studies on mouse ovaries have indicated that a reduction in the proportion of naive CD4 T cells is associated with a diminished ability to mount an immune response, potentially contributing to age-related reproductive decline (11). Furthermore, a wealth of evidence suggests that effector memory CD4 T cells play a vital role in maintaining fetal-maternal immune tolerance and preventing pregnancy loss (12). Circulating and regulatory NK cells represent two major subtypes of NK cells distinguished by their expression of CD56. CD56^bright^ NK cells, known as regulatory NK cells, generally constitute a small population and are recognized for their regulatory and immunomodulatory functions. Conversely, CD56^dim^ NK cells, referred to as circulating NK cells, are the predominant subset of NK cells and possess potent cytotoxic and effector functions. Although NK cells contribute to the innate immune system and aid in preserving the integrity of ovarian tissue by eliminating cells with abnormal growth or function, an excessive number of NK cells may lead to chronic inflammation and tissue damage (13). Previous evidence has identified that elevated levels of NK cells in the ovary can disrupt proper follicular development by exerting cytotoxic effects on granulosa cells, which are essential for follicular growth and maturation (14). These findings highlight the disruption of T cell reserves and NK cell activation during anovulation, potentially causing a homeostatic imbalance within the immune system. Therefore, it is crucial to investigate these immune alterations in ovulatory disorders to gain a deeper understanding of their underlying mechanisms.

In this study, we utilized single-cell RNA sequencing (scRNA-seq) to explore the immune landscape at high resolution in peripheral blood mononuclear cells (PBMCs) from patients with ovulation failure and compared them to healthy individuals. We identified a total of 25 distinct cell clusters and investigated the major changes that occurred in each cell type. Our findings revealed a significant disturbance in the NK and T cell populations in individuals with ovulation disorders. Our data also highlighted the crucial role of cDC-mediated cell-cell interactions, especially contributing to the amplification of global inflammatory responses in individuals with immune dysfunction. Furthermore, we observed a significant upregulation of oxidative stress in patients with ovulation disorders. In addition to enhancing our understanding of the immune mechanisms underlying ovulation dysfunction, the present study suggests potential therapeutic strategies for mitigating ovarian dysfunction in female reproductive health.

## Materials and methods

### Ethics statement and clinical sample collection

This study was approved by the Ethics Committee of the Department of Medical and Life Science, Tongji University, and written informed consent was obtained from each participant (2020tjdx067). All sample collections were strictly performed according to the ethical and biosafety protocols approved by the institutional guidelines.

Blood samples were collected from the Shanghai Tongji Hospital (China) between November 2020 and January 2021. The cohort included individuals with MENO (n = 2), PCOS (n = 3), and POI (n = 3) for subsequent 10× genomic scRNA-seq. To eliminate the influence of medication, all volunteers were sampled without drug administration. The diagnosis of menopause, PCOS, and POI was based on detailed clinical symptoms according to a previous guide (15–17). The menopausal ages of the two individuals in the menopausal control group were 51 and 52 years, respectively. They joined the study control group at 1 and 3 years after the onset of menopause. Both women experienced natural menopause and were excluded from any immune disorders. The detailed clinical information and demographic characteristics of the patient cohort are shown in **Table S1**. PBMCs sequencing data of healthy females (n = 3) were downloaded from the 10× genomics dataset (https://www.10xgenomics.com/resources/datasets).

### Single cell RNA library preparation and sequencing

PBMCs were isolated from whole blood samples according to the 10× Genomics Demonstrated Protocol (CG00039). Cell viability was assessed by trypan blue staining, and samples (cell viability >90%) were prepared using a 10× Genomics Single Cell 5’ v2 Reagent Kit according to the manufacturer’s instructions (10× Genomics). Each sequencing library was generated by using a unique sample index. The libraries were sequenced using an Illumina Nova6000.

### Single-cell RNA data pre-processing and analysis

The raw 5′ scRNA-seq data were processed using CellRanger software (version 6.1.1). The transcripts were aligned to the human reference genome h38 using the function of “cellranger count” with the default parameter. All processed data were input into the respective folder, and downstream analysis was performed.

Main analyses in downstream were performed on Seurat R package (version 4.0.10) (18). Cells with less than 300 detected genes were filtered. After data filtering, raw counts were normalized to 10,000 reads by “NormalizeData” function with the default parameters. By using “FindVariableFeatures” function with the method of “vst” and 2000 highly variable genes were identified to perform principal component analysis (PCA). By using function of “FindIntegrationAnchors” and “IntegrateData”, we reduced batch effect to reasonable degree and integrated the data from different batches to one Seurat object. The data were then scaled again using “ScaleData” and run a principal component analysis (PCA) and uniform manifold approximation and projection (UMAP) dimensionality reduction by using function of “RunPCA” and “RunUMAP.” A nearest-neighbor graph using 30 dimensions of the PCA reduction was calculated using ‘FindNeighbors,’ followed by clustering using ‘FindClusters’ with a resolution of 0.6. Gene ontology enrichment analysis was performed by the clusterProfiler R package (version 3.9.0) (19). Lineage scores were calculated according to a published article (20) by calculating the sum of the logarithm of cpms among selected genes (see **Table S2**). Venn diagrams were constructed using the Venn R package (version 1.10). Statistical analyses were performed by ggsignif R packages (version 0.6.0).

### Gene regulatory network analyses

To infer gene regulatory networks (GRNs) between fertile and infertile group, we used pySCENIC (version 0.12.0) (21) to perform regulatory networks analysis. The analysis steps consisted of three parts: creating a co-expression module *via* GRNBoost2, refining modules with cisTarget, and estimating the regulon activity by AUCell. The specific regulons among different groups (Health, MENO, PCOS and POI) were identified using the Regulon Specificity Score (RSS) according to standard tutorials. Gene regulatory networks were visualized with customization by cytoscape (version 3.16.1).

### Receptor and ligand interactions analysis

To assess the cell–cell interactions and significant pathways, we performed all cell matrix from different group on CellChat R packages (version 1.1.3) (22). For put data into CellChat, we extracted large dgCMatrix from Seurat objects by using function of “GetAssayData.” Briefly, we followed official supported workflow and loaded normalized data into CellChat by function of “createCellChat.” We used CellChatDB in human secreted signaling as a ligand–receptor interaction database for subsequent analysis. Then, by using ‘computeNetSimilarityPairwise’ function, we identified signaling between healthy and POI. Differential expression of signaling pathway between healthy and POI was identified by function of ‘identifyOverExpressedGenes.’ To explore selected signaling pathway among different cell types, we ranked similarity of the shared signaling pathways by applying function of ‘rankSimilarity.’

### Statistical analysis

The bioinformatics data were statistically analyzed using ordinary one-way ANOVA or wilcox test with Graphpad Prism (version 9.4.1) and P < 0.05 was considered statistically significant. In this paper, one, two and three asterisks indicate p < 0.05, p < 0.01 and p < 0.001 respectively.

## Results

### Study design and single immune cell profiling among individuals with ovulation dysfunction

**Figure 1:**
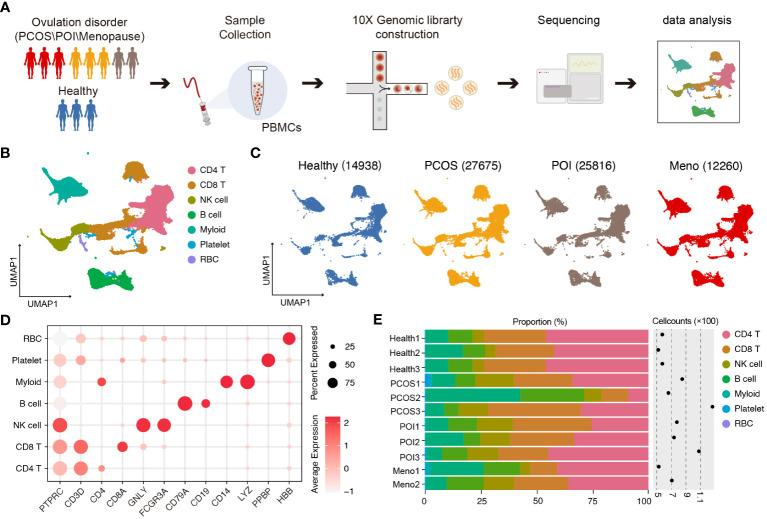
Single-cell transcriptomic profiles of PBMCs in ovulation-dysfunctional and healthy females. **(A)** Overview of sample collection, sequencing, and downstream analyses. **(B)** UMAP plot of the scRNA-seq profiled dataset for seven major cell types. **(C)** UMAP plot of the distribution of single cells among the different groups. **(D)**. Violin plots showing marker genes for diverse immune cell subsets. **(E)** Bar plot showing the percentage of major cell types in PBMCs of each individual. The cell counts for each sample are listed on the right-hand side.

To characterize the immune properties of females who have experienced ovulation dysfunction, we generated an scRNA-seq dataset consisting of eight infertile females, including two females with menopause (MENO), three patients with polycystic ovary syndrome (PCOS), three patients with primary ovarian insufficiency (POI), and a control group of three healthy females (**Figure 1A**). Using the uniform manifold approximation and projection (UMAP) technique, we analyzed the distribution of various cell lineages in peripheral blood mononuclear cells (PBMCs) based on the expression of canonical cell markers. We observed that major cell types were present in all groups (**Figures 1B, C**). Specifically, we identified seven major cell clusters: CD4 T cells (CD3D, CD4), CD8 T cells (CD3D, CD8), natural killer (NK) cells (GNLY, CD16), B cells (CD79A, CD19), myeloid cells (CD14, LYZ), platelets (PPBP), and red blood cells (RBC) (**Figure 1D**). The distribution patterns of these major cell populations were comparable across patient groups (PCOS/POI/MENO) (**Figure 1E**).

### Cellular characterization of individuals with ovulation disorder

**Figure 2:**
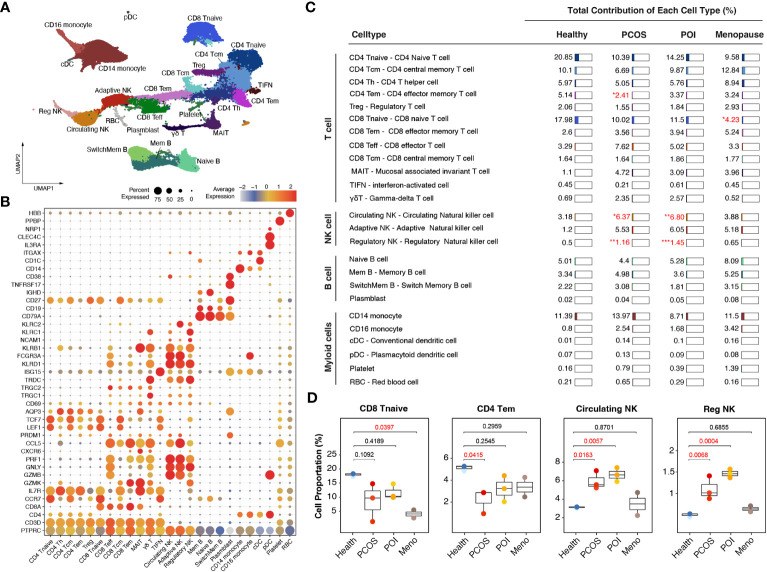
Immunological alterations in patients with ovulation disorders. **(A)** UMAP plot showing all the identified cells from ovulated and healthy females. **(B)** Violin plots showing special markers for all sub-cell types. **(C)** Table showing the percentages of each cell type among different groups (health/menopause/PCOS/POI). Ovulation-dysfunctional and healthy groups were expressed as percentages of the total immune cell types. **(D)** Boxplot showing the proportions of cells with significant differences in each sample colored by individual. The x-axis corresponds to each patient group. Significant differences compared to control samples were calculated by ordinary one-way ANOVA.

Based on graph-based clustering of uniform manifold approximation and projection (UMAP) and specific gene markers, our data identified 25 cell subtypes, including CD4 naive T cells, CD4 central memory T cells, CD4 T helper cells, CD4 effector memory T cells, Treg, CD8 naive T cells, CD8 effector memory T cells, CD8 effector T cells, CD8 central memory T cells, mucosal associated invariant T cells, interferon-activated T cells, gamma-delta T cells, circulating NK cells, adaptive NK cells, regulatory NK cells, naive B cells, memory B cells, switch memory B cells, plasmblasts, CD14 monocytes, CD16 monocytes, conventional dendritic cells (cDCs), plasmacytoid dendritic cells (pDCs), platelets, and red blood cells (RBCs) (**Figures 2A, B**).

Specifically, within the CD4-positive T cell population, we distinguished CD4 naive T cells (marked by CCR7, LEF1, and TCF7), CD4 central memory T cells (characterized by high CCR7 expression and increased AQP3 and CD69 compared to CD4 naive T cells), CD4 effector memory T cells (identified by PRDM1 and low CCR7 expression), Tregs (expressing IL2RA and FOXP3), and CD4 Th1 cells (marked by CXCR3). In the CD8-positive T cell population, we identified CD8 naive T cells (marked by CCR7, LEF1, and TCF7), CD8 central memory T cells (showing high CCR7 expression and increased AQP3 and CD69 compared to CD8 naive T cells), CD8 effector memory T cells (characterized by GZMK expression), CD8 effector T cells (exhibiting high levels of GZMB, GNLY, and PRF1 but lacking CCR7 and IL7R expression), and mucosal-associated invariant T cells (MAIT) (marked by GZMK and high IL7R expression). Additionally, we identified CD4 and CD8 negative T cell subtypes, including gamma-delta T cells (expressing TRGC1, TRGC2, and TRDC) and interferon-activated cells (TIFN) (identified by ISG15 expression) (**Figure 2B**). We observed three distinct sub-clusters within the NK cell population: circulating NK cells (expressing FCGR3A and exhibiting dim NCAM1 expression), adaptive NK cells (marked by KLRC2 and dim NCAM1 expression), and regulatory NK cells (expressing FCGR3A, bright NCAM1, and KLRC1) (**Figure 2B**). We identified four different subtypes of B cells based on their gene expression profiles. Switched memory B cells expressed CD19 and CD27 but lacked IGHD expression, naive B cells expressed IGHD but lacked CD27 expression, memory B cells expressed both CD27 and IGHD, mature B cells expressed CD19 and MS4A1 but lacked CD27 expression, and plasmablasts expressed high levels of TNFRSF17 and CD27. Additionally, we identified CD14 monocytes (expressing CD14), CD16 monocytes (expressing FCGR3A), cDCs (marked by CD1C and ITGAX expression), and pDCs (expressing IL3RA, CLEC4C, and NRP1) (**Figure 2B**).

Comparing these cell subtypes across the anovulation groups with healthy females, we observed decreased proportions of CD4 naive T cells, CD8 naive T cells, and CD4 effector memory T cells, whereas all NK cell subtypes showed an increase (**Figures 2C**, **S1**) in the ovulation-dysfunction group. Specifically, the number of CD8 naive T cells significantly decreased in the MENO group, whereas CD4 effector memory T cells significantly decreased in the PCOS group (**Figure 2D**). The percentages of circulating and regulatory NK cells exhibited dramatic increases in the PCOS and POI groups (**Figure 2D**).

### cDC-mediated cell–cell communication tends to disorder in ovulation-dysfunction groups

**Figure 3:**
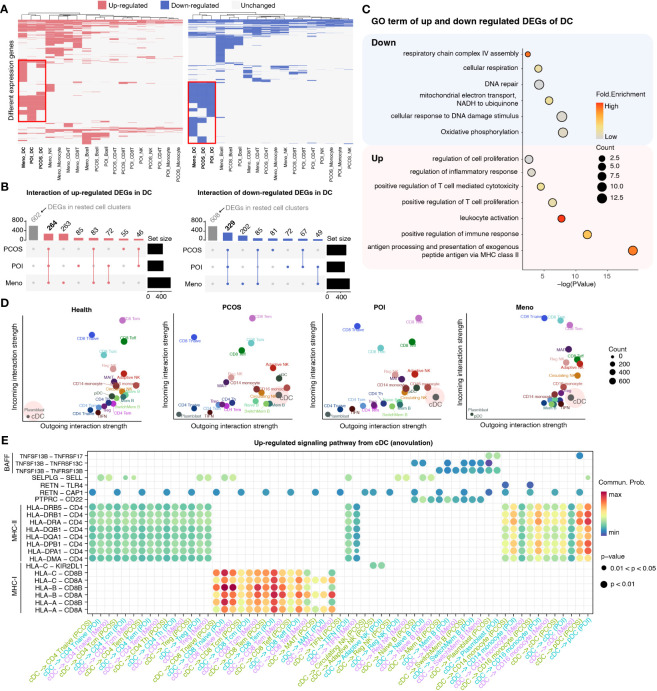
Detailed characterization of DCs in each ovulation-dysfunction group. **(A)** Heatmaps showing the distribution of DEGs between PCOS, POI, Meno, and Health groups in major cell subtypes. The red box shows the differential genes of DC cells by comparing PCOS, POI, and Meno to Health. **(B)** Upset plot showing upregulated (left) and downregulated (right) DEGs in DC. **(C)** The representative GO terms of upregulated and downregulated DEGs overlapped among PCOS, POI, and Meno in DCs. **(D)** A scatter plot of the outgoing and incoming interaction strengths identified significant changes in sending or receiving signals among diverse cell types in each group. **(E)** Overview of communication probabilities mediated by ligand–receptor pairs from cDCs to rested cell types significantly increased in the anovulatory groups.

To investigate specific gene expression alterations for ovulation dysfunction, we conducted a detailed comparison of gene expression in five major cell types, CD4 T cells, CD8 T cells, NK cells, dendritic cells (DCs), and monocytes, between the ovulation-dysfunction group and the healthy group (**Figure 3A**). Interestingly, we observed that DCs exhibited the highest number of differentially expressed genes (both upregulated and downregulated) among the three ovulation-dysfunction groups, suggesting a potential key role for DCs in immune alterations associated with ovulation dysfunction (**Figure 3A**). Through interaction plots, we discovered that most of the differentially expressed genes were shared among the ovulation groups, with 264 common genes being upregulated and 329 common genes being downregulated (**Figure 3B**). Gene ontology (GO) analysis revealed that the upregulated genes were enriched for MHC-II antigen processing and presentation and regulation of immune cell activation, while the downregulated genes were enriched for oxidative phosphorylation and cellular respiration (**Figure 3C**).

To examine whether changes in DC function were related to the dynamics of NK cell subpopulations, we explored the strength of the interaction among different cell populations between the healthy and ovulation-dysfunction groups using the CellChat R package (**Figure S2**). The results demonstrated that the interaction strength sourcing from cDCs was significantly increased in all ovulation-dysfunction groups compared to that in the healthy group (**Figures 3D**, **S2**). Furthermore, to elucidate the specific signaling pathways involved in cDC-mediated cell–cell communication, we compared the communication probabilities mediated by ligand–receptor pairs from cDCs to almost other cell populations. Our findings revealed a predominant presence of signaling pathways involving MHC-I, MHC-II, and BAFF, which are known to play a significant role in promoting inflammatory responses in cDC-mediated cell–cell communication (**Figure 3E**). We found that the ligand–receptor pair of HLA-DRB5-CD4, HLA-DRB1-CD4, HLA-DRA-CD4, HLA-DQB1-CD4, HLA-DQA1-CD4, HLA-DPB1-CD4, HLA-DPA1-CD4, and HLA-DMA-CD4 significantly contributed to communication from cDCs to CD4 T cells and monocytes. HLA-A-CD8A/B, HLA-B-CD8 A/B, HLA-C-CD8 A/B significantly influenced communication from cDCs to CD8 T cells and TNFSF13B-TNFRSF13B/C significantly affected communication between cDCs and B cells (**Figure 3E**). These results collectively indicate that cDC may play an essential role in the progression of inflammation during ovulation dysfunction.

### Expression dynamics show abnormal immune activation in ovulation disorder

**Figure 4:**
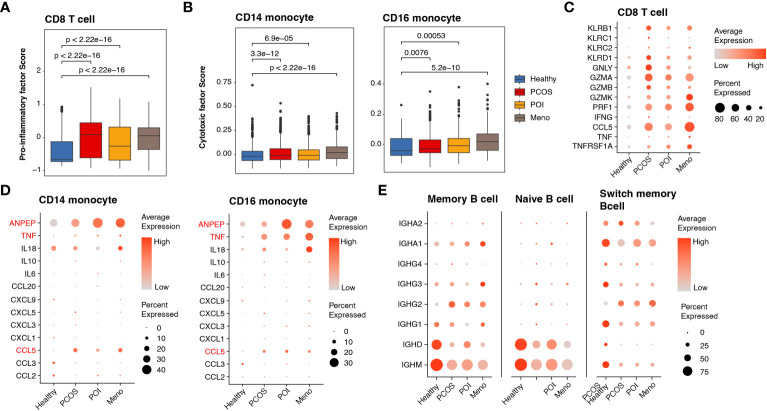
Abnormally activated cell cytotoxins and inflammatory response in ovulation dysfunction. **(A, B)** Box plot showing the lineage score of **(A)** pro-inflammatory factors in CD8 T cells and **(B)** cytotoxic mediators in both CD14 and CD16 monocytes and the different groups. **(C, D)** Dotplot depicting the expression of detailed genes for calculating the **(C)** pro-inflammatory factor score in CD8 T cells and **(D)** cytotoxic mediator score in CD14/CD16 monocytes among different groups. **(D)** Dotplot showing the expression of genes involved in immunoglobulin in the B cells of each group.

**Figure 5:**
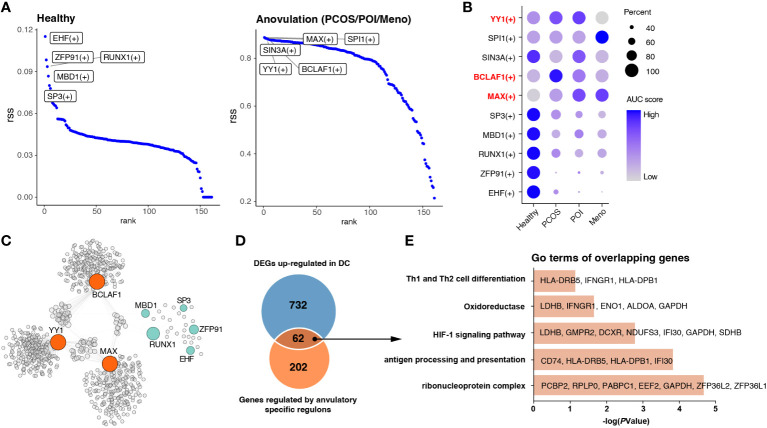
Identification of key regulons of DCs in ovulation disorders. **(A)** Rank of regulons in cDCs between healthy subjects and others (PCOS, POI, and MENO) based on the Regulon Specificity Score (RSS). The top-ranked regulon activities are shown in the picture. **(B)** Dotplot showing the AUC score for each regulon in each group. **(C)** Network of selected regulons and their target genes in group of ovulation-dysfunction and healthy group. **(D)** Venn diagram showing the overlapping genes that were upregulated in DCs and regulated by ovulation-specific regulons in **(C)**. **(E)** Bar plots of the representative GO terms of the overlapping genes.

Given the substantial upregulation of pro-inflammatory pathways mediated by cDCs, we investigated alterations in pro-inflammatory factors among major inflammation-related cell types, such as CD8 T cells, monocytes, and B cells. To better estimate functional dynamics comprehensively, we calculated the lineage score according to published articles (20). Compared with the healthy group, all ovulation-dysfunction groups showed a significant increase in pro-inflammatory factors in CD8 T cells (**Figure 4A**). Furthermore, we observed a significant increase in cytotoxic factors in CD14 and CD16 monocytes in the ovulation-dysfunction groups (**Figure 4B**). These findings indicated the presence of aggravated inflammatory responses and immune disorders during the progression of ovulation dysfunction. To investigate which genes contributed to these immune changes, we examined the expression of all genes used to calculate the lineage score in **Figures 5C, D** across different cell types. Our analysis revealed substantial upregulation of numerous pro-inflammatory factors, including KLRB1, KLRD1, GZMA, GZMB, GZMK, PRF1, CCL5, and TNFRSF1A, in CD8 T cells in all ovulation-dysfunctional groups (**Figure 4C**). Compared to healthy individuals, pro-inflammatory cytokines such as ANPEP, TNF, and CCL5 were significantly elevated in both CD14 and CD16 monocytes of ovulation-dysfunction individuals (**Figure 4D**). It is well established that immunoglobulin class switching plays a crucial role in effective immune responses by allowing the immune system to adapt antibody production to different types of pathogens and immune challenges (23). Our data indicated that B cells in the healthy group predominantly expressed IGHD/IGHM, whereas B cells in the ovulation-dysfunction groups mainly exhibited IGHA and IGHG. Together, our data revealed that CCL5 largely contributes to ovulation-related inflammation and indicates that the immunoglobulin class switch from IGHD/IGHM to IGHA/IGHG contributes to chronic inflammation during ovulation dysfunction.

### Gene regulatory network analyses revealed key regulators involving in immune changes of cDC among ovulation-dysfunction patients

In view of the significant alterations in gene cluster expression in cDCs, we considered that some transcription factors may be the main regulators resulting in immunological changes. All cDC were subjected to SCENIC analysis to construct the gene regulatory networks. Our results identified essential regulons among the four groups, and the top five most-active regulons in the healthy and each ovulation-dysfunction group are shown in **Figure 5A**. When considering the rank score, we observed that *EHF*, *ZFP91*, *RUNX1*, *MBD1*, and *SP3* were specifically upregulated in the healthy group, whereas *YY1*, *BCLAF1*, and *MAX* were specifically upregulated in the ovulation-dysfunction groups (**Figures 5A, B**). Using the eight regulons mentioned above, we constructed predicted regulatory networks (**Figure 5C**, **Table S3**). By mapping these networks with 794 unique upregulated DEGs in DCs (**Figure 3A**), an overlap of 62 genes, representing nearly 30% of the DEGs, was identified (**Figure 5D**). To gain further insight, we performed gene ontology (GO) analysis of these overlapping genes. The DEGs were enriched in functions such as antigen processing and presentation, Th1 and Th2 cell differentiation, and the HIF-1 signaling pathway (**Figure 5E**). These findings suggest that *YY1*, *BCLAF1*, and *MAX* play pivotal roles in immune changes in cDCs and cDC-mediated inflammatory response pathways. Overall, these results shed light on the transcriptional regulatory landscape of cDCs in the context of blood ovulation-dysfunction, highlighting the activation of specific transcription factors during immunological changes.

### Global oxidative stress enhances in ovulation disorder

**Figure 6:**
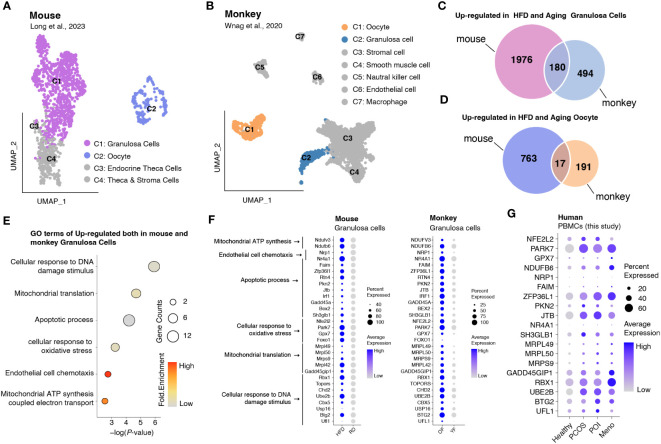
Integrated data analysis revealed that aberrant oxidative stress occurs in both granulosa cells and PBMCs during ovulation disorders. **(A)** UMAP plot of all identified cells from the HFD and RD mice. **(B)** UMAP plot of all the identified cells from young and aged monkeys. **(C)** Venn diagram showing the upregulation in HFD and aging granulosa cells. **(D)** Venn diagram showing the upregulation in HFD and aging oocytes. **(E)** Dotplot depicting representative GO terms of genes upregulated in both mouse and monkey granulosa cells. **(F,G)** Dotplot showing the expression of genes involved in representative GO terms in **(F)** granulosa cells of mice and monkeys separately and **(G)** PBMCs of humans in each group.

Analysis of DEGs revealed that in most PBMC cell types, genes involved in reducing oxidative stress, such as *JUN* and *JUND*, were significantly downregulated, whereas some genes associated with mitochondrial respiration, such as *MT-ATP8* and *MT*-*ND4L*, exhibited high expression levels (**Figure S3**). To assess whether increased oxidative stress is widely present during ovulation, we obtained data from monkeys and mice and investigated gene expression changes in oocytes and granulosa cells in high-fat mice and aged monkeys (24, 25) (**Figures 6A, B**). A comparison between oocytes and granulosa cells revealed a higher number of upregulated DEGs in the latter, including genes shared between mice and monkeys (**Figures 6C, D**). Gene ontology (GO) analysis demonstrated that the commonly upregulated DEGs in granulosa cells of both mice and monkeys were primarily associated with cellular responses to oxidative stress and DNA damage stimulus, mitochondrial translation, apoptotic process, and cell chemotaxis (**Figure 6E**). We further presented the expression profiles of DEGs involved in these pathways as a dot plot (**Figure 6F**) and observed that many of the genes involved in oxidative stress and mitochondrial energy metabolism (such as *NDUFV3*, *NDUFB6*, *NFE2L2*, *PARK7*, and *GPX7*), mitochondrial translation (such as *MRPL49*, *MRPL50*, and*MRPS9*), DNA damage stimulus response (*GADD45GIP1*, *RBX1*, *UBE2B*, *BTG2*, and *UFL1*), and apoptosis (*ZFP36L1*, *PKN2*, and *JTB*) that were highly expressed in granulosa cells were also highly expressed in human PBMCs (**Figure 6G**).

## Discussion

This study investigated the immunological changes in patients with anovulation, including PCOS, POI, and menopause. Our findings suggest that aberrant changes in T and NK cell populations, augmented inflammatory responses mediated by cDCs, and global oxidative stress in PBMCs are common characteristics of ovarian dysfunction. Importantly, these aberrations may pose a high risk for long-term chronic inflammation and have detrimental effects on ovarian function. In summary, this study provides valuable insights into the immunological changes associated with anovulation, shedding light on the potential mechanisms and implications of therapeutic interventions.

Although long-term inflammatory reactions have been reported to potentially damage tissues and cells, affecting their function and structure (26), our study provides essential insights into the inflammatory response in various types of anovulation. CD8 T cells, CD14 monocytes, and CD16 monocytes are known to play critical roles in both cytotoxic and pro-inflammatory responses, contributing to immune defense against infections and regulation of inflammatory processes in different disease contexts (27, 28). Our findings demonstrated that a hyperinflammatory phenotype is significantly enhanced during anovulation, characterized by the upregulation of pro-inflammatory factors such as CCL5 in CD8 T cells and CD14/CD16 monocytes. Furthermore, we observed a shift in immunoglobulin production from IgD/IgM to IgA/IgG during anovulation. Previous research has suggested that IgA and IgG are two proinflammatory immunoglobulins (29), and immunoglobulin isotypes such as IgG can interact with Fc gamma receptors on immune cells to regulate their effector functions, including inflammation (30). These insights significantly advance our understanding of chronic inflammation in ovulatory disorders, emphasizing the crucial role of increased cytokine production from CD8+ T cells and monocytes, global oxidative stress, and immunoglobulin switching in inflammatory mediation. Targeting of these processes has the potential to alleviate chronic inflammation and restore normal ovulatory function.

Conventional dendritic cells (cDCs) play a crucial role in inflammation by interacting with various immune cells including CD4 T cells, CD8 T cells, B cells, and monocytes (31). They influence immune responses through cell–cell signaling and cytokine production, particularly *via* MHC-I, MHC-II, and BAFF pathways (31). CD8 T cells, also known as cytotoxic T cells, recognize antigens presented by MHC-I molecules and release cytotoxic molecules and proinflammatory cytokines upon activation (32). Monocytes, through MHC-II expression, scan for foreign antigens and initiate proinflammatory responses to modulate inflammation (33). Additionally, BAFF pathway activation can induce class switching in B cells, leading to the production of specific immunoglobulin isotypes, such as IgG and IgA, which are important for immune responses and inflammation (34). Our data showed that abnormal upregulation of the MHC-I, MHC-II, and BAFF pathways by cDCs was activated during anovulation, which is likely to intensify the inflammation response. Additionally, transcription factors such as YY1, BCLAF1, and MAX, are enriched in cDCs from women with ovulation disorders and are involved in antigen presentation. Abnormal activation of these factors impairs cDC immune activity. Targeting YY1, BCLAF1, and MAX could be a potential therapeutic strategy to restore cDC functionality and reduce inflammation during anovulation. Overall, the increased inflammatory response in women with ovulation disorders is directly linked to cDC-mediated immune signaling.

Previous studies have established that oxidative stress can provoke immune dysfunction by affecting immune cell function, cytokine and chemokine production, and the promotion of inflammation (35). In our study, we observed a consistent reduction in the expression of oxidative stress-related genes, including *JUN* and *FOS*, in almost all cellular clusters of peripheral blood mononuclear cells (PBMCs). Given that granulosa cells serve as the energy source for oocytes, abnormal levels of oxidative stress can affect oocyte development, potentially leading to ovulation disorders (36, 37). Supplementing our findings with transcriptome data derived from the ovaries of high-fat diet-induced mice and aging monkeys, we identified a concurrent upregulation of oxidative stress-related genes, specifically *NDUFV3*, *NDUFB6*, *NFE2L2*, *PARK7*, and *GPX7* (24), in both granulosa cells and PBMCs of women suffering from ovulatory disorders. Additionally, genes associated with DNA damage stimulus response (*GADD45GIP1*, *RBX1*, *UBE2B*, *BTG2*, and *UFL1*) and apoptosis progress (*ZFP36L1*, *PKN2*, and *JTB*) concurrently increase in response to oxidative stress were observed in our data. These findings imply that the irregular variations in oxidative stress levels in peripheral blood parallel those observed in granulosa cells across mammals, and aberrant oxidative stress may exacerbate DNA damage and cellular apoptosis. Together, our study revealed heightened oxidative stress, characterized by an imbalance between the production of reactive oxygen species (ROS) and antioxidant defense mechanisms, among patients with anovulation. This phenomenon could potentially serve as a valuable biological marker for ovulation disorders.

In conclusion, our study provides a comprehensive comparative analysis of the common types of ovulation disorders, revealing significant immune alterations in affected women. These alterations include an elevated inflammatory response and oxidative stress. Importantly, our findings highlight the central role of the cDC-centered signaling pathway in driving the excessive inflammatory response observed during anovulation. Consequently, targeting this pathway, as well as reducing oxidative stress and modulating CD8+ T cell and NK cell activity, presents a promising approach for enhancing immune function and restoring normal ovarian function in patients with ovulation disorders. However, it is crucial to acknowledge the limitations of our study, particularly its small sample size. Therefore, further independent validation using techniques such as flow cytometry and additional functional experiments are necessary to confirm and strengthen these findings. Addressing these limitations will improve the reliability and significance of future research in this field.

## Data availability statement

The datasets presented in this study can be found in online repositories. The names of the repository/repositories and accession number(s) can be found below: https://ngdc.cncb.ac.cn/, HRA003535.

## Ethics statement

The studies involving humans were approved by the Ethics Committees of Department of Medical and Life Science, Tongji University. The studies were conducted in accordance with the local legislation and institutional requirements. The participants provided their written informed consent to participate in this study.

## Author contributions

BL: Project administration, Supervision, Writing – original draft, Writing – review & editing. LQ: Conceptualization, Data curation, Investigation, Methodology, Software, Writing – original draft, Writing – review & editing. YL: Data curation, Formal Analysis, Resources, Writing – review & editing. LZ: Resources, Writing – review & editing. SL: Resources, Writing – review & editing. XZ: Resources, Writing – review & editing. WL: Formal analysis, Writing – review & editing. JQ: Formal Analysis, Writing – review & editing. XC: Investigation, Writing – review & editing. YJ: Funding acquisition, Project administration, Supervision, Writing – review & editing. ZX: Funding acquisition, Project administration, Supervision, Writing – review & editing.

## Supplementary material

Supplementary Figure 1 Statistics of identified cell percentage in each group. Boxplot showing the percentages of all cell populations among different groups (Health/MENO/PCOS/POI).

Supplementary Figure 2 Overview of cell-cell communications.Heatmap displaying details of the differential interaction strength of health vs MENO(left), health vs PCOS (middle) and health vs POI (right). The right bar plot showing the sum of outgoing signaling. For color bar, red means increased signaling and blue means decreasing signaling in the second dataset comparing with the first one.

Supplementary Figure 3 Intersection of up-regulated and down-regulated DEGs of each group in major cell types. **(A, B)** Upset plot showing up-regulated **(A)** and down-regulated **(B)** DEGs among B cells, monocytes and dendritic cells between health and MENO, health and PCOS and health and POI separately. **(C, D)** Upset plot showing up-regulated **(A)** and down-regulated **(B)** DEGs among CD4 T cells, CD8 T cells and NK cells between health and MENO, health and PCOS and health and POI separately.

Supplementary Table 1 The clinical data statistics of patients with ovulation disorder.

Supplementary Table 2 Gene list showing interaction of DEGs of DCs in each group.

Supplementary Table 3 Tables of DC-special TFs and their targeted genes.
