# Supplementary figures and images for: Immune and oxidative stress disorder in ovulation-dysfunction women revealed by single-cell transcriptome
Source: Front Immunol. 2023 Dec 4;14:1297484. doi: 10.3389/fimmu.2023.1297484 (PMC10729704; doi:10.3389/fimmu.2023.1297484)

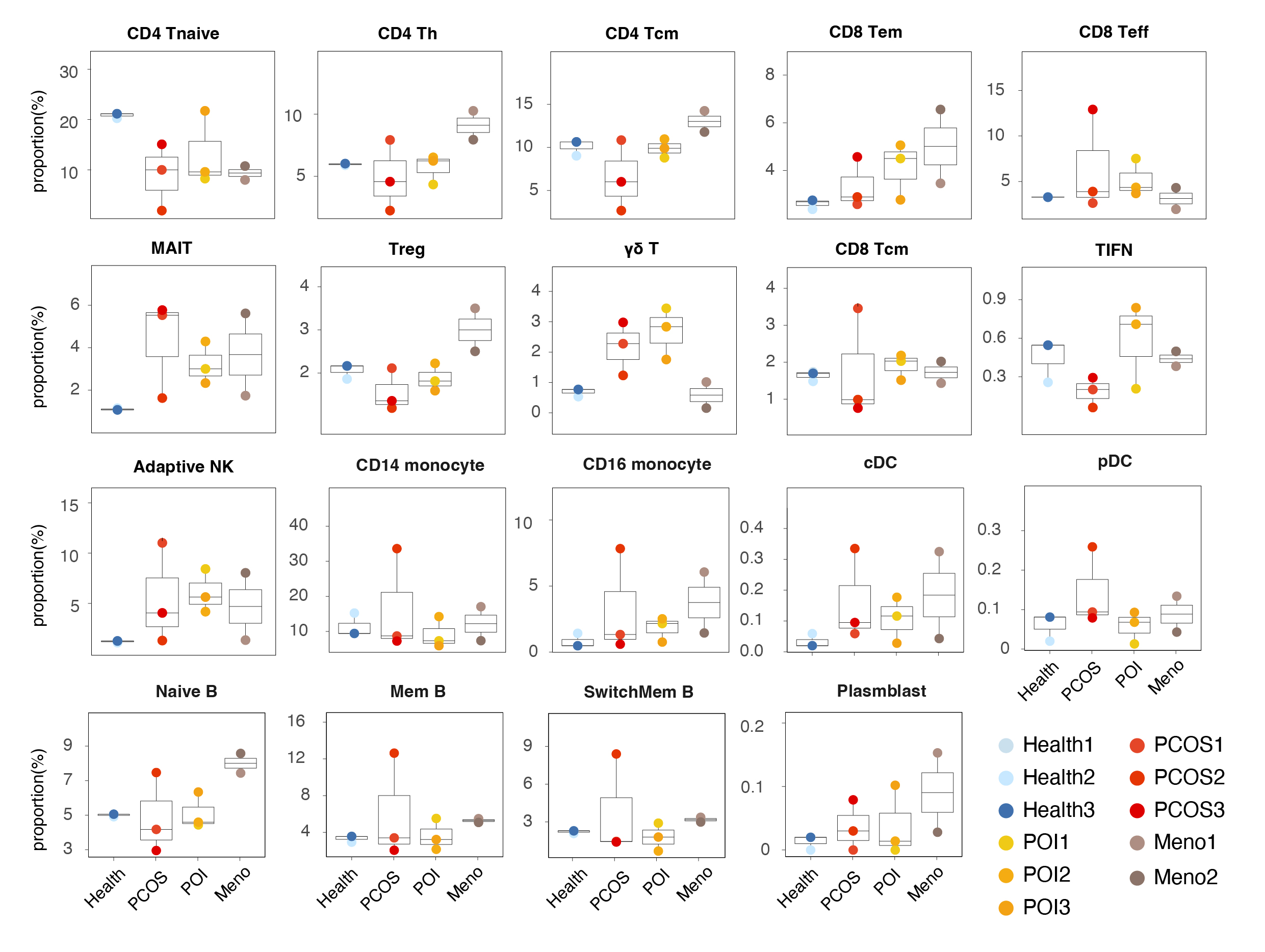

Supplement: Supplementary Figure 1 — Statistics of identified cell percentage in each group. Boxplot showing the percentages of all cell populations among different groups (Health/MENO/PCOS/POI). [file Image_1.jpeg]

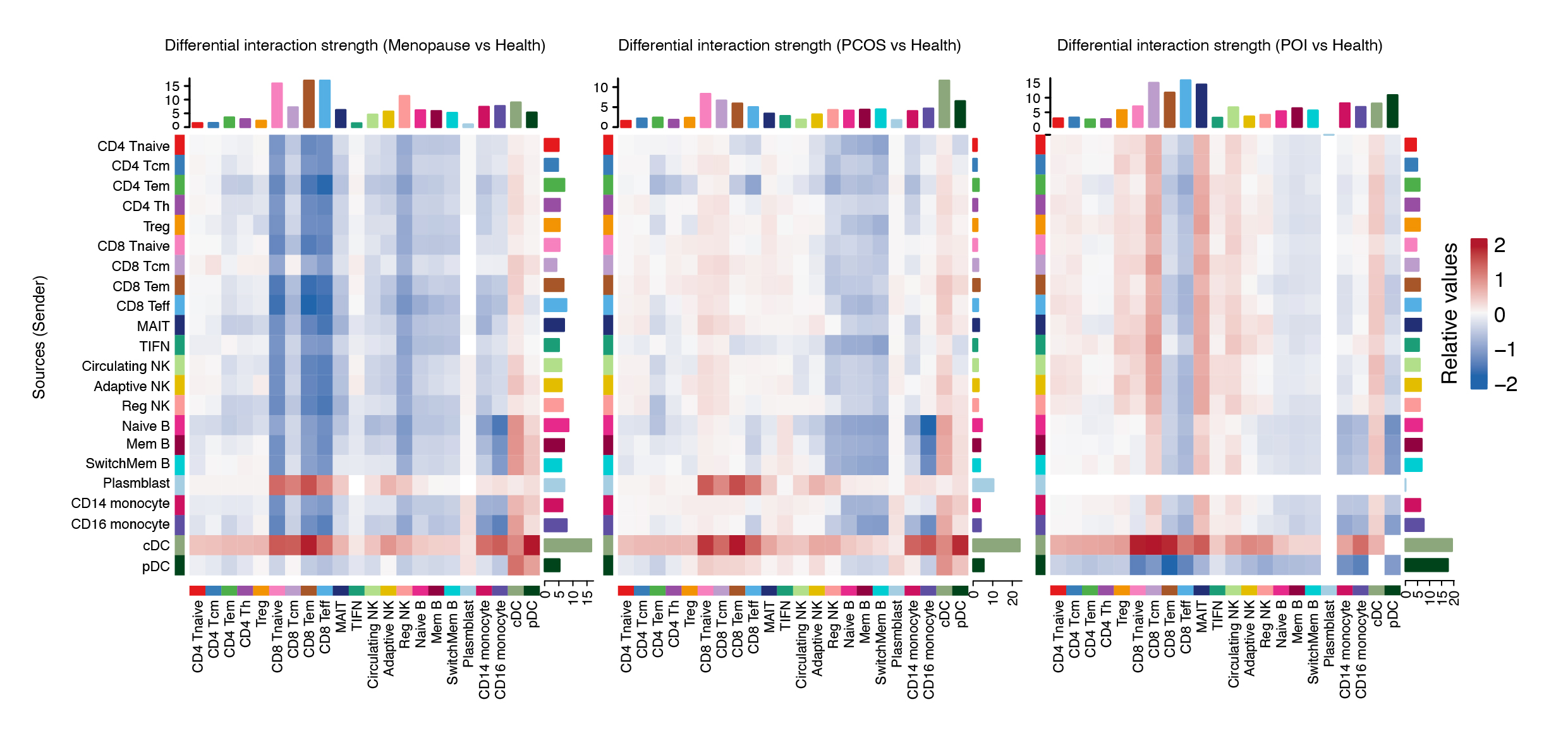

Supplement: Supplementary Figure 2 — Overview of cell-cell communications.Heatmap displaying details of the differential interaction strength of health vs MENO(left), health vs PCOS (middle) and health vs POI (right). The right bar plot showing the sum of outgoing signaling. For color bar, red means increased signaling and blue means decreasing signaling in the second dataset comparing with the first one. [file Image_2.jpeg]

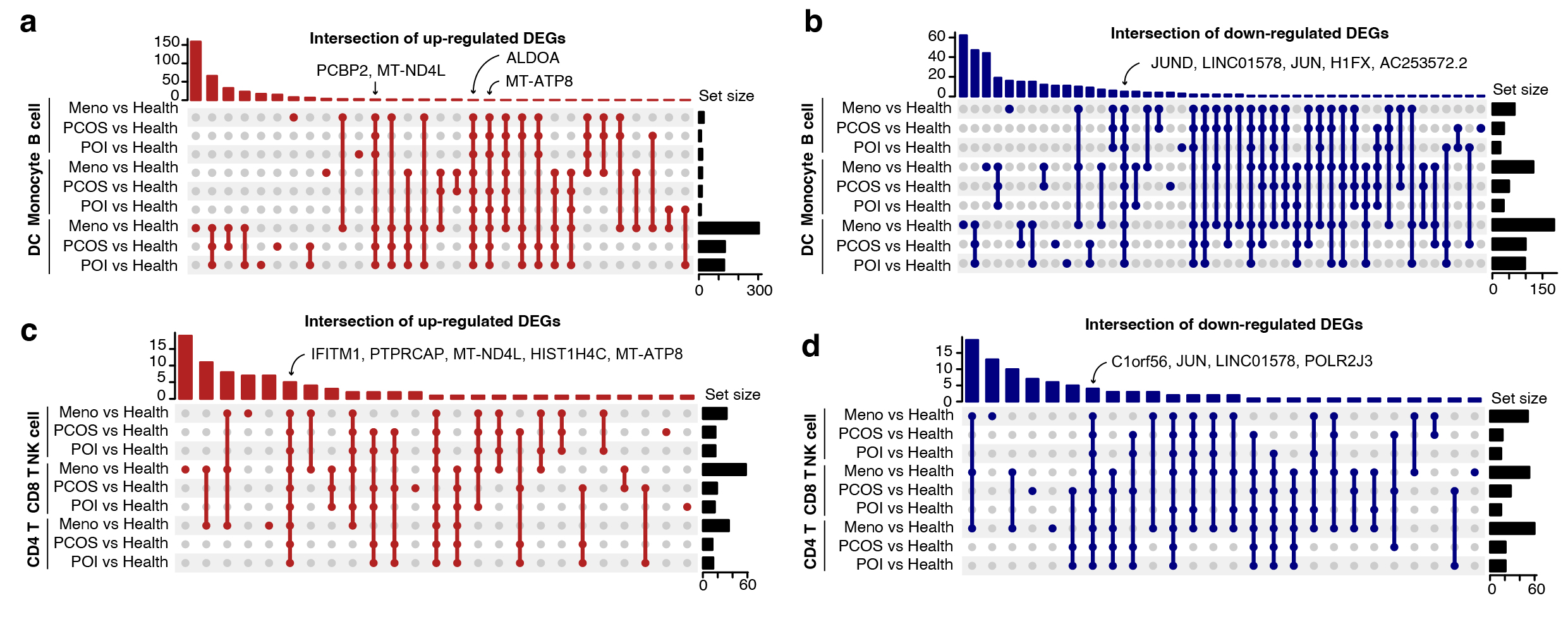

Supplement: Supplementary Figure 3 — Intersection of up-regulated and down-regulated DEGs of each group in major cell types. (A, B) Upset plot showing up-regulated (A) and down-regulated (B) DEGs among B cells, monocytes and dendritic cells between health and MENO, health and PCOS and health and POI separately. (C, D) Upset plot showing up-regulated (A) and down-regulated (B) DEGs among CD4 T cells, CD8 T cells and NK cells between health and MENO, health and PCOS and health and POI separately. [file Image_3.jpeg]
